# Diagnostic Performance of QFR for the Evaluation of Intermediate Coronary Artery Stenosis Confirmed by Fractional Flow Reserve

**DOI:** 10.21470/1678-9741-2018-0234

**Published:** 2019

**Authors:** Zhenhua Xing, Junyu Pei, Jiabing Huang, Xinqun Hu, Shan Gao

**Affiliations:** 1 Department of Cardiology, Second Xiangya Hospital, Changsha, China.; 2 Department of Geriatrics, Second Xiangya Hospital,Changsha, China.

**Keywords:** Myocardial Fractional Flow Reserve, Coronary Artery Disease, Quantitative Flow Ratio, Sensitivity and Specificity, Meta-Analysis [Publication Type]

## Abstract

**Introduction:**

Quantitative flow ratio (QFR) is a novel method enabling efficient computation of FFR from three-dimensional quantitative coronary angiography (3D QCA) and thrombolysis in myocardial infarction (TIMI) frame counting. We decided to perform a systematic review and quantitative meta-analysis of the literature to determine the correlation between the diagnosis of functionally significant stenosis obtained by QFR versus FFR and to determine the diagnostic accuracy of QFR for intermediate coronary artery stenosis.

**Methods:**

We searched PubMed, Embase, and Web of Science for studies concerning the diagnostic performance of QFR. Our meta-analysis was performed using the DerSimonian and Laird random effects model to determine sensitivity, specificity, positive likelihood ratio (LR+), negative likelihood ratio (LR-), and diagnostic odds ratio (DOR). The sROC was used to determine diagnostic test accuracy.

**Results:**

Nine studies consisting of 1175 vessels in 1047 patients were included in our study. The pooled sensitivity, specificity, LR+, LR-, and DOR for QFR were 0.89 (95% CI: 0.86-0.92), 0.88 (95% CI: 0.86-0.91), 6.86 (95% CI,: 5.22-9.02), 0.14 (95% CI: 0.10-0.21), and 53.05 (95% CI: 29.75-94.58), respectively. The area under the summary receiver operating characteristic (sROC) curve for QFR was 0.94.

**Conclusion:**

QFR is a simple, useful, and noninvasive modality for diagnosis of functional significance of intermediate coronary artery stenosis.

**Table:** 

Abbreviations, acronyms & symbols				
3D	= Three-dimensional		LR+	= Positive likelihood ratio
3D QCA	= Three-dimensional quantitative coronary angiography		LR	= Negative likelihood ratio
AUC	= Area under the curve		OR	= Odds ratio
CI	= Confidence interval		MI	= Myocardial infarction
DOR	= Diagnostic odds ratio		PCI	= Percutaneous coronary intervention
EAPCI	= European Association of Percutaneous Cardiovascular Interventions		PRISMA	= Preferred Reporting Items for Systematic Review and Meta-Analysis Protocols
ESC	= European Society of Cardiology		QFR	= Quantitative flow ratio
FFR	= Fractional flow reserve		QUADAS-2	= Quality Assessment of Diagnostic Accuracy Studies 2
FP	= False positive		sROC	= Summary receiver operating characteristic
FAVOR	= Functional Diagnostic Accuracy of Quantitative Flow Ratio in Online Assessment of Coronary Stenosis study		STEMI	= ST-elevation myocardial infarction
FN	= False negative		TIMI	= Thrombolysis in myocardial infarction
LR	= Likelihood ratio		TN	= True negative
			TP	= True positive

## INTRODUCTION

Accurate evaluation of coronary artery disease, especially intermediate coronary artery stenosis, is crucial for the evaluation of myocardial ischemia and next treatment. The gold standard for diagnosis and confirmation of functional significance of a stenosis is the fractional flow reserve (FFR). Previous studies have demonstrated that FFR-guided coronary revascularization increases the ratio of event-free survival when compared with a coronary stenosis-guided strategy^[1,2]^. Despite these advantages, the clinical application of FFR has been variable and slow^[3]^. FFR requires not only the hyperemic state, but also additional cost, time, and efforts.

Quantitative flow ratio (QFR) is a novel method enabling efficient computation of FFR from three-dimensional quantitative coronary angiography (3D QCA) and thrombolysis in myocardial infarction (TIMI) frame counting^[4]^. Compared with FFR, QFR does not require any invasive physiological measurements, pharmacological hyperemia induction, and additional cost. The recently FAVOR (Functional Diagnostic Accuracy of Quantitative Flow Ratio in Online Assessment of Coronary Stenosis) II China study showed solid results for QFR computation in identifying the presence of functionally significant stenosis in eligible patients^[5]^. Several studies have been published in the literature addressing the correlation between the assessment of functionally significant stenosis obtained by QFR versus FFR and addressing the diagnostic accuracy of QFR for intermediate coronary artery stenosis^[5,6]^. The purpose of our study was to perform a systematic review and quantitative meta-analysis of the literature to determine the correlation between the diagnosis of functionally significant stenosis obtained by QFR versus FFR, and to determine the diagnostic accuracy of QFR for intermediate coronary artery stenosis.

## METHODS

This protocol is reported following the Preferred Reporting Items for Systematic Review and Meta-Analysis Protocols (PRISMA) guidelines^[7]^. We searched PubMed, Embase, and Web of Science published before March 15, 2018. The keywords used for search were "QFR or Quantitative flow ratio". Results were limited to trials published in English. We manually searched reference lists of relevant studies and reviews, editorials, and letters to identify further articles. We used Endnote (Thompson ISI ResearchSoft, Philadelphia, USA) to manage relevant articles and remove duplicated articles.

### Study Eligibility

The inclusion criteria for studies in the analysis were as follows: 1) The design was a diagnostic accuracy study; 2) The study assess the diagnostic performance of QFR compared with invasive FFR as the standard procedure; 3) Data from true positive (TP), false positive (FP), true negative (TN), false negative (FN), sensitivity and specificity can be retrieved or calculated. When relevant data were missing, authors were contacted by e-mail, before excluding the study due to inaccessibility of data.

### Data Collection and Quality Assessment

Relevant data were initially extracted by two independent reviewers (Zh Xing and Jy Pei). Disagreements were resolved by consensus or by a third investigator (XQ Hu). We abstracted the following data from the selected articles: first author, publication date, study design, patient demographics; FFR threshold used to describe ischemia; and the data of TP, FP, TN, and FN. When different flow models of QFR were performed, contrast-flow QFR was preferred. Contrast-flow QFR was more accurate for predicting FFR ≤0.80 as compared with fixed-flow QFR^[4]^. Included studies were analyzed by the Quality Assessment of Diagnostic Accuracy Studies 2 (QUADAS-2)^[8]^.

### Data Analysis

The inter-reviewer agreement regarding the quality assessment of included studies was assessed by the Cohen kappa test. Our meta-analysis was performed using the DerSimonian and Laird random effects model to determine sensitivity, specificity, positive likelihood ratio (LR+), negative likelihood ratio (LR), and diagnostic odds ratio (DOR). The sROC was used to determine diagnostic test accuracy. An area under the curve (AUC) between 0.75 and 0.92 represented a high degree of diagnostic accuracy, and an AUC between 0.93 and 0.96 was considered more accurate. In order to assess heterogeneity among the studies, the^[2]^ statistic was used. For^[2]^, a value >50% was considered of severe heterogeneity. The Spearman correlation coefficient was calculated to evaluate diagnostic threshold variation among the included studies.

We also performed a meta-regression analysis to identify predefined potential sources of heterogeneity. All statistical analyses were completed using Meta-DiSc (version 1.4).

## RESULTS

### Study Selection and Characteristics

**Fig. 1:**
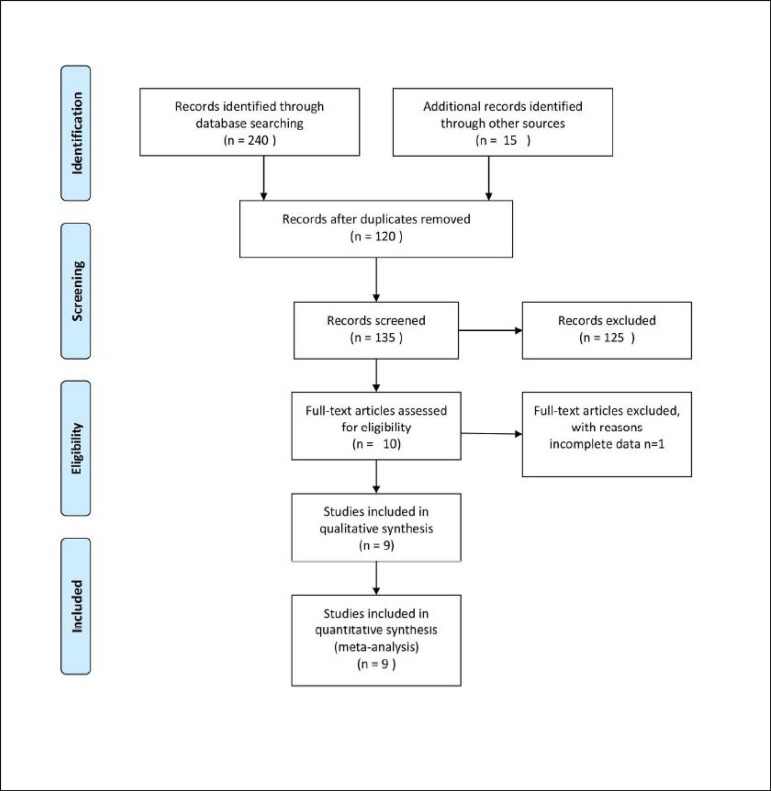
Flowchart for the identification of studies.

The flowchart of our search and selection process was presented in Figure 1. Our combined search strategy identified possible relevant studies. Nine studies were retrieved for a more detailed evaluation. Finally, 9 studies consisting of 1175 vessels in 1047 patients met our inclusion criteria^[4-6^^), (^^9-15]^. Characteristics of included studies were shown in Table 1. Clinical heterogeneity was mostly due to different inclusion criteria. Mejia-Renteria^[9]^ and Emori^[13]^ included patients with myocardial infarction. Emori^[13]^ included two different populations: patients with previous myocardial infarction (MI) and patients without previous MI. Spitaleri^[12]^ included patients with ST- elevation myocardial infarction (STEMI) and multivessel disease. Four studies were performed in Japan, two in China, one each in Spain, Italy, and Netherlands. The mean (SD) age was 63.2 years, and 68.1% of the patients were male. The quality assessment of included studies according to QUADAS-2 was presented in Supplementary Figure 1. In general, there was low risk of bias and low concern regarding applicability of all included studies.

**Table 1:** Characteristics of included studies.

Study	Design	Country	Mean age (years)	Males (%)	Patients' characteristics	Cutoff
Emori et al.^[15]^	Multicenter	Japan	-	-	Intermediate stenosis with FFR	0.8
Xu et al.^[5]^	Prospective, multicenter	China	61	73.7	Intermediate stenosis with FFR	0.8
Yazaki et al.^[6]^	Retrospective, single-center	Japan	72.5	29.6	Intermediate stenosis with FFR	0.8
Emori et al.^[13]^ MI*	Retrospective, single-center	Japan	69	83	Patients with previous MI undergoing CAG and FFR	0.8
Emori et al.^[13]^ non-MI*	Retrospective, single-center	Japan	70	54	Intermediate stenosis with FFR	0.8
Tu et al.^[4]^	Prospective, multicenter	China	65.8	61	Intermediate stenosis with FFR	0.8
Kameyama et al.^[11]^	Multicenter	Japan	-	-	ACS patients with CAG and FFR	0.8
van Rosendael et al.^[10]^	Prospective, single-center	Netherlands	64	71	Intermediate stenosis with FFR	0.8
Mejia-Renteria et al.^[9]^	Multicenter	Spain	-	-	Patients with CAG and FFR	0.8
Spitaleri et al.^[12]^	Prospective, multicenter	Italy	62	28	STEMI patients with MVD and FFR	0.8
Spitaleri et al.^[12]^	Prospective, multicenter	Denmark	61	67	Intermediate stenosis with FFR	0.8

* Emori 2018 contained two groups: patients with previous myocardial infarction (MI) and patients with no previous MI.

ACS=acute coronary syndrome; CAG=coronary angiography; FFR=fractional flow reserve; MVD=multivessel disease; STEMI=ST-elevation myocardial infarction

### Diagnostic Accuracy of QFR

The results of the included study were presented in Table 2. The accuracy ranged from 80% in Kameyama^[11]^ and 94% in Spitaleri, et al.^[12]^. Sensitivity ranged between 74% in Tu, et al.^[4]^ and 100% in van Rosendael, et al.^[10]^ and the specificity ranged from 79% in Emori, et al.^[15]^ to 97% in Spitaleri, et al.^[12]^. The correlation between QFR and FFR ranged from r = 0.69 to r = 0.94.

**Table 2:** Results of included studies in these meta-analyses.

Study	Included vessels (n)	Sensitivity (%)	Specificity (%)	Accuracy (%)	Correlation (r)
Emori et al.^[15]^	73	82	79	81	0.69
Xu et al.^[5]^	328	94.6	91.7	92.7	0.86
Yazaki et al.^[6]^	151	88.7	89.1	88.7	0.84
Emori et al.^[13]^ MI	75	92	82	87	0.88
Emori et al.^[13]^ non-MI	75	95	88	92	0.94
Tu et al.^[4]^	84	74	91	86	0.77
Kameyama et al.^[11]^	25	80	80	80	0.63
van Rosendael et al.^[10]^	15	100	79	80	0.78
Mejia-Renteria et al.^[9]^	300	88	86	87	-
Spitaleri et al.^[12]^	49	88	97	94	0.90

MI=myocardial infarction

**Fig. 2:**
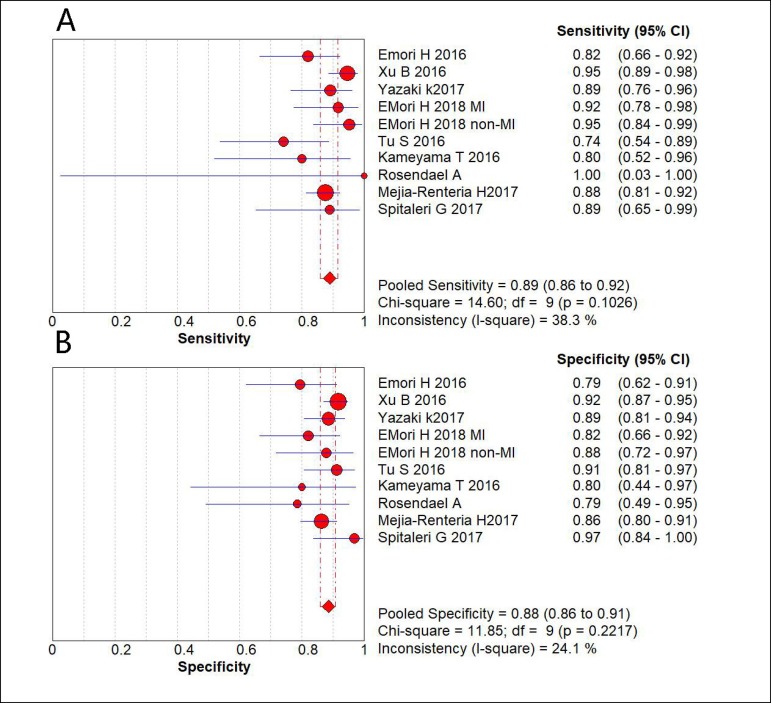
Forest plot of the sensitivity and specificity of included study, summary sensitivity and specificity and I2 statistic for heterogeneity.

**Fig. 3:**
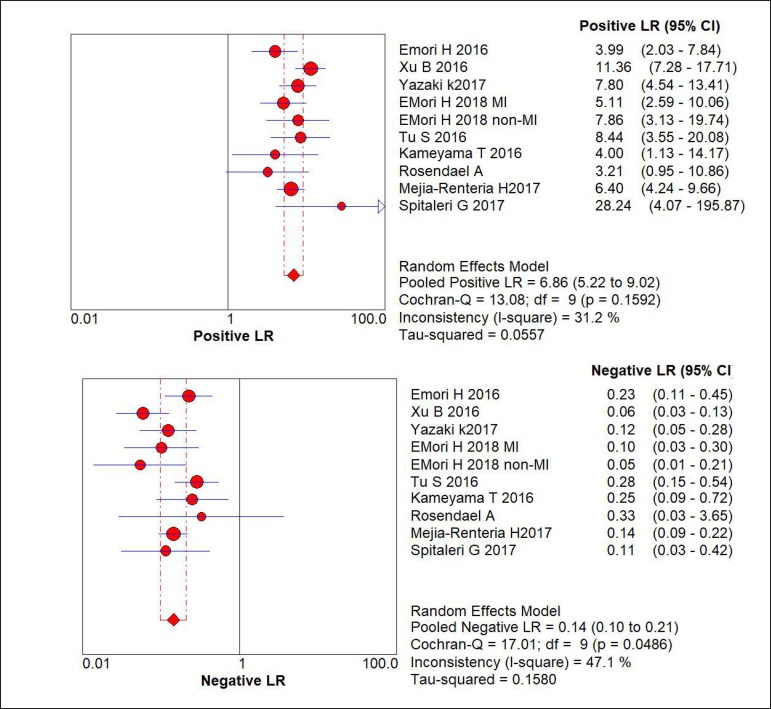
Forest plot of LR+ and LR- of included study, summary sensitivity and specificity, and I2 statistic for heterogeneity.

**Fig. 4:**
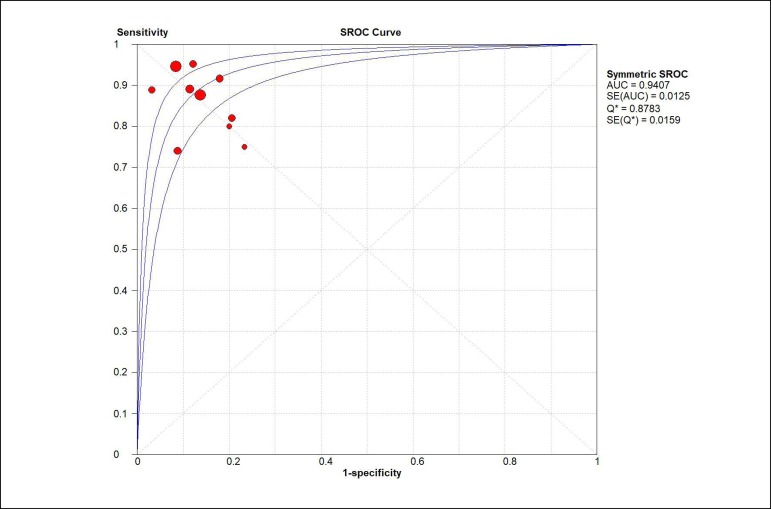
Summary receiver operating characteristic (sROC) curve for QFR.

In pooled data weighted by the number of vessels, QFR had a combined sensitivity and the specificity of QFR for diagnosis of functional significance of a stenosis according to FFR were 0.89 (95% CI: 0.86-0.92) and 0.88 (95% CI: 0.86-0.91), respectively, using a random effects model (Figure 2). No heterogeneity was found for both sensitivity (I^2^=38.3%, *P*=0.10) and specificity (I^2^=24.1%, *P*=0.22). The pooled estimate of positive likelihood ratio (LR+) and negative likelihood ratio (LR-) was 6.86 (95% CI: 5.22-9.02) and 0.14 (95% CI: 0.10-0.21) (Figure 3). For QFR, Spearman's correlation coefficients were 0.619 (*P*=0.102), indicating that the diagnostic threshold effect did not exist in QFR data. The area under the ROC curve was 0.94 (Figure 4) and the diagnostic OR was 53.05 (95% CI: 29.75-94.58) (Supplementary Figure 2).

### Meta-regression Analysis and Subgroup Analysis

Meta-regression was performed using the potential sources of heterogeneity among studies (age, country, sex, different inclusion criteria). We found no factor effecting the diagnostic accuracy.

Emori, et al.^[13]^ and Mejia-Renteria, et al.^[9]^ included patients with previous MI that might affect the diagnostic accuracy. Exclusion of these two trials slightly improved the specificity (0.90, 0.87-0.92), but did not affect sensitivity.

## DISCUSSION

In the present study, we performed a systematic review of the diagnostic performance of QFR for functional significance of intermediate coronary artery stenosis compared with invasive FFR. Data from 1175 vessels in 1047 patients showed the situation in which QFR is helpful for surgeons to determine whether stents should be implanted at no additional cost, time, and effort. Our study is the first systemic review and meta-analysis that evaluates the diagnostic accuracy of QFR for the assessment of functionally significant stenosis confirmed by FFR.

FFR-guided percutaneous coronary intervention (PCI) is associated with a better outcome compared with revascularization based on angiographic stenosis severity alone in patients with intermediate coronary artery stenosis^[2,16,17]^. FFR has the highest recommendation (class I, level A) in the European Society of Cardiology guideline on myocardial revascularization^[18]^. Although FFR has better outcomes in patients with intermediate coronary artery stenosis, the clinical application has been limited due to the invasive procedure with a pressure wire, the cost of pressure wire, and the side effects associated with induction of hyperemia. QFR, an angiographic index of coronary stenosis severity based on 3D QCA and thrombolysis in myocardial infarction (TIMI) frame counting, estimates FFR without invasive procedure^[4]^. FAVOR Pilot Study and FAVOR II China study have shown good agreement of QFR with invasive FFR^[4,5]^. However, the simple size was too small. Our meta-analysis included^[9]^ studies of which the majority were performed in China and Japan from 2016 to 2018, with a total of 1175 vessels in 1047patients, and demonstrated that the diagnostic accuracy of QFR for functionally significant stenosis confirmed by FFR was high, with a summary sensitivity and specificity of 0.89 (95% CI: 0.86-0.92) and 0.88 (95% CI: 0.86-0.91), respectively.

Our studies included different study populations (stable coronary artery disease, suspected coronary artery disease, STEMI, previous MI). However, our meta-regression showed that different study populations did not affect our diagnostic accuracy. QFR was more often used in patients with stable coronary artery disease. A recent study has found that QFR may be a safe and reliable tool to guide revascularization in patients with STEMI and multivessel disease. Furthermore, Spitaleri found that functional complete revascularization evaluated by QFR showed a good 5-year outcome^[12]^.

However, the diagnostic accuracy of QFR for assessing the functional severity of coronary stenosis might be affected in coronary arteries related to previous MI^[13]^. Microcirculatory resistance may affect this phenomenon. Mejia-Renteria, et al. found that the diagnostic accuracy of QFR was lower in patients with high microcirculatory resistance, which is supported by our subgroup analysis. Due to its specific algorithm based on QCA and TIMI frame counting, coronary collateral circulation may reduce its accuracy. Therefore, corrective measures need to be developed to improve the diagnostic accuracy in patients with previous MI or high microcirculatory resistance. Furthermore, coronary calcification or thrombus may lead to angiographic haziness which undoubtedly reduces the diagnostic accuracy of QFR. A hybrid QFR-FFR approach may be a way to overcome these limitations. Yazaki, et al.^[6]^ found that FFR should be performed in stenosis with QFR 0.75-0.85. This hybrid approach may allow clinicians to get the best of both worlds by ensuring diagnostic accuracy while reducing cost and side effects.

It should be noted that our conclusion should be seen in the context of its limitation. First, the simple size is relatively small. Second, although there was no apparent heterogeneity in statistics, the heterogeneity in clinical and methodology was inevitable.

## CONCLUSION

QFR is a simple, useful, and noninvasive modality for the diagnosis of functional significance of intermediate coronary artery stenosis.

**Table:** 

Authors' roles & responsibilities	
ZX	Designed the study and provided methodological expertise; final approval of the version to be published
JP	Drafted the manuscript; final approval of the version to be published
JH	Drafted the manuscript; final approval of the version to be published
XH	Drafted the tables and figures; final approval of the version to be published
SG	Designed the study and provided methodological expertise; final approval of the version to be published
